# Length-Dependent Translation Efficiency of ER-Destined Proteins

**DOI:** 10.3390/cimb45080425

**Published:** 2023-08-14

**Authors:** Hana Sahinbegovic, Alexander Vdovin, Renata Snaurova, Michal Durech, Jakub Nezval, Jiri Sobotka, Roman Hajek, Tomas Jelinek, Michal Simicek

**Affiliations:** 1 Faculty of Medicine, University of Ostrava, Syllabova 19, 703 00 Ostrava, Czech Republic; 2 Department of Hematooncology, University Hospital Ostrava, 17. listopadu 1790/5, 708 00 Ostrava, Czech Republic; 3 Faculty of Science, Department of Physics, University of Ostrava, 30. dubna 22, 701 03 Ostrava, Czech Republic; 4 Laboratory of Medical Genetics, SPADIA LAB a.s., 700 30 Ostrava, Czech Republic

**Keywords:** proteosynthesis, mRNA, signal peptide, endoplasmic reticulum, ribosome stalling

## Abstract

Gene expression is a fundamental process that enables cells to produce specific proteins in a timely and spatially dependent manner. In eukaryotic cells, the complex organization of the cell body requires precise control of protein synthesis and localization. Certain mRNAs encode proteins with an N-terminal signal sequences that direct the translation apparatus toward a specific organelle. Here, we focus on the mechanisms governing the translation of mRNAs, which encode proteins with an endoplasmic reticulum (ER) signal in human cells. The binding of a signal-recognition particle (SRP) to the translation machinery halts protein synthesis until the mRNA–ribosome complex reaches the ER membrane. The commonly accepted model suggests that mRNA that encodes a protein that contains an ER signal peptide continuously repeats the cycle of SRP binding followed by association and dissociation with the ER. In contrast to the current view, we show that the long mRNAs remain on the ER while being translated. On the other hand, due to low ribosome occupancy, the short mRNAs continue the cycle, always facing a translation pause. Ultimately, this leads to a significant drop in the translation efficiency of small, ER-targeted proteins. The proposed mechanism advances our understanding of selective protein synthesis in eukaryotic cells and provides new avenues to enhance protein production in biotechnological settings.

## 1. Introduction

Translation is a fundamental and tightly regulated process used by every cell to produce proteins by decoding the genetic information from the form of an mRNA transcript into an amino acid sequence in a polypeptide. A single transcript can be read by multiple ribosomes at the same time, forming a complex known as a polysome [1]. Once engaged, the polysome will translate the mRNA and will not readily exchange it for newly added mRNA until the translation of the initial mRNA is completed [2]. Transcripts associated with multiple ribosomes suggest robust translation. On the other hand, mRNAs with low or no ribosome associations are expected to be translated poorly or remain untranslated [3]. Additionally, the presence of rare codons, complex secondary structures, premature stop codons, or missense sequences can result in ribosome collisions and stalling events during the translation elongation phase [4]. Subsequently, the inhibition of translation triggers ribosome quality control and mRNA surveillance pathways that ultimately lead to the dissociation of ribosomes and the degradation of nascent peptides [5].

The endoplasmic reticulum (ER) plays a vital role in the correct assembly of proteins destined for membrane integration or secretion. ER-directed mRNA encompasses about 30–40% of all eukaryotic transcripts [6]. The majority of these mRNAs do not bind to ER itself and their association with ER is enabled by the binding interactions of the ribosome and translational product. The best-described mechanism for the ER localization of mRNA relies on the presence of a specific stretch of amino acids known as a signal peptide (SP), which, upon translation, is recognized by a signal recognition particle (SRP) [7,8].

During translation, polypeptides are translocated into the ER lumen, which significantly differs from the cytosol in terms of ion concentration and redox conditions. Importantly, the ER lumen promotes a variety of post-translational modifications and chaperone-facilitated folding, and is thus crucial for the proper assembly and function of most of the membrane and secretory proteins [9]. Proteins detected as terminally misfolded are destined for degradation by the cytosolic ubiquitin–proteasome system [10]. Moreover, protein quality control in the ER stops protein synthesis while simultaneously expanding the capacity for folding and degradation in response to stress [11]. Implementing strict control measures in the ER guarantees the capture of potentially harmful proteins [12].

In this study, we investigate the length-dependent translation efficiency of the ER-destined SP-containing proteins encoded by mRNAs in human cells. We found that the protein synthesis into the ER is significantly less efficient in short mRNAs containing SP compared to longer mRNAs. The apparent translation deficiency for shorter, ER-localized mRNAs is not affected by the components of the ribosome stalling pathway. We also show that cells tend to upregulate the expression of shorter transcripts; however, this does not fully compensate for a drop in protein synthesis. Furthermore, our work suggests that mRNAs encoding short proteins with ER-localization SPs are less associated with the ER membrane and excluded from polysome fractions. Altogether, we propose a model that suggests the translation mechanism for short and long ER-bound protein-encoding transcripts.

## 2. Materials and Methods

### 2.1. Cell lines, Culture Conditions and Transfections

HEK293 cells were maintained in Dulbecco’s Modified Eagle’s Medium (DMEM; high glucose, pyruvate and L-glutamine) containing 10% fetal bovine serum (FBS) and 1% penicillin/streptomycin and maintained in a 5% CO_2_ humidified incubator. Cells were transiently transfected with the respective plasmids using polyethyleneimine (PEI; 1 mg/mL) and serum-free media (Opti-MEM), mixed and incubated for 15 min at RT when cells were at 60% confluence 24 h after seeding.

### 2.2. Reporter Design

Translation efficiency reporters were designed as previously described [13]. Briefly, the reporter cassette contains N- and C-terminal GFP and RFP markers separated by an analyzed sequence. We analyzed Ig domains (one and two domains) from an Ig light chain isolated from the RPMI8226 cell line, as well as Ig-like domains from Filamin A. The domains were linked together using a VLRQPKAA linker derived from the original light chain molecule. The analyzed sequence is flanked by viral P2A sequences, at which ribosomes skip the formation of a peptide bond without interrupting translation elongation. Complete translation of the cassette will generate three proteins in equal amounts. By contrast, translation interruption before RFP synthesis results in a sub-stoichiometric RFP:GFP ratio.

### 2.3. Quantitative Real-Time PCR

QIAzol Lysis Reagent (Qiagen, Hilden, Germany) was used to extract total RNA. The RNA samples’ quality (purity and integrity) was determined utilizing an Agilent 2100 Bioanalyzer with an RNA 600 NanoLabChip reagent set (Agilent Technologies, Santa Clara, CA, USA). The RNA was quantified via Qubit fluorometry (Thermo Scientific, Waltham, MA, USA). A RevertAid First Strand cDNA Synthesis Kit (Thermo Scientific) was used for complementary DNA (cDNA) synthesis according to the manufacturer’s instructions. Quantitative PCR was conducted using PowerUpTMSYBRTM Green Master Mix (Applied Biosystems, Waltham, MA, USA) on a StrepOnePlus real-time PCR system (Applied Biosystems). Relative mRNA expression was calculated using the 2-DDCt method and normalized to GAPDH transcripts.

### 2.4. Flow Cytometry Analysis

Translation efficiency analysis employing flow cytometry was performed using the stalling reporter system as described previously [13]. Briefly, 10^6^ HEK293 cells transfected with a dual-fluorescent reporter containing (K)0 or (K^AAA^)20, or a reporter with one, two, or four domains of IgL; or one, two, or four domains of FLNA; or one domain of IgL with ZNF598, were trypsinized after 72 h of transfection using TrypleX, sedimented (600× *g* for 3 min), washed in PBS, sedimented again and resuspended in PBS containing SYTOX blue, and analyzed using a Beckman Coulter CytoFLEX S instrument (Beckman Coulter Inc., Brea, CA, USA). Approximately 10^4^ events were collected in the SYTOX-negative gate for each sample. Geometric mean statistics were used to analyze MFI via FlowJo v10 software.

### 2.5. SDS-PAGE and Western Blotting

The cell lysates were resolved using SDS PAGE and transferred to the polyvinylidene difluoride (PVDF) membrane. Membranes were blocked in 5% (*w*/*v*) nonfat milk (Roth) in PBS-T (phosphate-buffered saline, 0.05% Tween 20) and incubated with the appropriate primary antibodies overnight at 4 °C in 1% (*w/v*) BSA/PBS-T. Primary antibodies used at the indicated dilutions included anti-GFP (MMS-118P, Biolegend (San Diego, CA, USA), 1:2000), anti-RPS10 (sc-515655, Santa Cruz (Dallas, TX, USA), 1:1000) and anti-Ub (3936, Cell Signaling Technology (Danvers, CA, USA), 1:1000). Membranes were subsequently washed with PBS-T and incubated with horseradish peroxidase (HRP)-conjugated secondary antibody goat anti-mouse-IgG (115-035-146, Jackson ImmunoResearch (Tucker, GA, USA), 1:2000) for 1 h at room temperature. Signal detection was performed using ECL (Thermo Fisher Scientific) and a ChemiDoc MP System (Bio-Rad, Hercules, CA, USA). Protein band analysis was conducted via densitometry utilizing ImageJ v1.49 (National Institutes of Health, Bethesda, MD, USA).

### 2.6. Preparation of Cell Lysates for Ribo Mega-SEC

We used the protocol developed in [14]. Briefly, a total of 2 × 10^7^ HEK293 transfected with the reporter system were treated with 50 µg/mL cycloheximide to maintain polysome stability for 5 min under 37 °C and 5% CO_2_ before harvest. Cells were then washed with ice-cold PBS containing 50 µg/mL cycloheximide, lysed by vortexing for 10 s in 400 µL of polysome extraction buffer (20 mM HEPES-NaOH, pH 7.4, 130 mM NaCl, 10 mM MgCl_2_, 1% CHAPS, 2.5 mM DTT, 50 µg/mL cycloheximide, 20 U RNase inhibitor murine—New England BioLabs; EDTA-free protease inhibitor cocktail—Thermo Scientific) and incubated for 30 min on ice. Insoluble material was pelleted via centrifugation at 17,000× *g* for 10 min at 4 °C. The supernatant was filtered through 0.45 µm Ultrafree-MC HV centrifugal filter units at 12,000× *g* for 2 min, and the total RNA amount in the filtrate was quantified via Qubit fluorometry (Thermo Scientific).

### 2.7. Polysome Separation Using Ribo Mega-SEC

An Agilent Bio SEC-5 column (2000 Å, 7.8 × 300 mm, 5 µm particles) together with an Agilent Bio SEC-5 guard column (2000 Å, 7.8 × 50 mm, 5 µm particles) were connected to an Agilent 1200 HPLC system (Agilent Technologies) and equilibrated with 2 column volumes (CV) of filtered SEC buffer (20 mM HEPES-NaOH, pH 7.4, 100 mM NaCl, 10 mM MgCl_2_, 0.3% CHAPS, 2.5 mM DTT). A total of 100 µL of 10 mg/mL of filtered bovine serum albumin (BSA) solution diluted using SEC buffer was injected once to block the sites for nonspecific interactions. After monitoring the column condition by injecting 25 µL of GeneRuler 1 kb DNA Ladder (Thermo Scientific), 100 µL of cell lysate containing 120 µg of RNA was loaded on the column. All column conditioning and separation were carried out at 12 °C. The chromatogram was monitored by measuring UV absorbance at 215, 260 and 280 nm using a diode array detector. The flow rate was 0.8 mL/min.

### 2.8. Fractionation via Sequential Detergent Extraction

Fractionation of the HEK293 cells was performed according to the protocol used by Jagannathan et al., 2011 [15]. Briefly, HEK293 cells at 80–90% confluency were treated with ice-cold PBS containing 50 µg/mL cycloheximide for 10 min, followed by incubation with 1 mL of ice-cold permeabilization buffer for 5 min. Soluble material was collected as the cytosolic fraction. Cells were then washed with ice-cold wash buffer and incubated with 1 mL of ice-cold lysis buffer for 5 min. The soluble fraction was collected as a membrane fraction. Both cytosolic and membrane fractions were centrifuged at 7500× *g* for 10 min and clear supernatants were transferred to new tubes.

### 2.9. Databases

#### Statistical Analysis

The statistical significance of differences between various groups was calculated using the two-tailed unpaired *t*-test. Error bars represent the standard deviation of the mean (SD). Statistical analyses, unless otherwise indicated, were performed using GraphPad Prism version 5.

## 3. Results

### 3.1. Single IgL Domain Decreases Translation Efficiency Similarly to Poly-A Sequence

**Figure 1:**
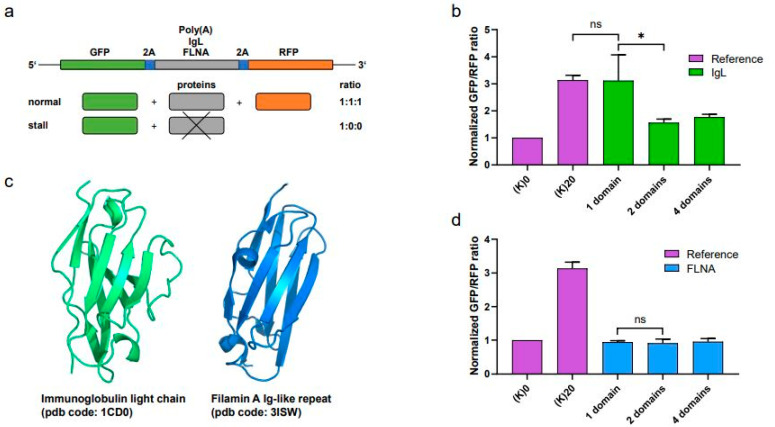
Single Ig domain abrogates translation. (**a**) Schematic representation of the translation efficiency reporter system with various stalling cassettes. (**b**) Normalized GFP:RFP fluorescence ratio of the translation efficiency reporter containing the control (K)0 and stalling (K)20 cassettes (reference, purple) and repeats of 1, 2 and 4 IgLλ domains (IgL, green). Significance was compared using the two-tailed Student’s *t*-test; * = *p*, 0.05. Error bars represent the mean ± SD. (**c**) X-ray structures of IgLλ (green, PDB code: 1CD0) and Filamin A (blue, PDB code: 3ISW). (**d**) Normalized GFP:RFP fluorescence ratio of the translation efficiency reporter containing the control and stalling cassettes (K)0 and (K)20 (reference, purple) and repeats of 1, 2 and 4 FLNA Ig-like domains (FLNA, blue). Significance was compared using the two-tailed Student’s *t*-test; * *p* = 0.05, ns = non-significant. Error bars represent the mean ± SD.

To date, ribosome stalling has been mainly associated with mRNA pathologies such as the presence of premature stop codons or complex secondary structures. Several studies have also suggested the need for translation slowdown in the interdomain regions to allow for correct protein folding. This might increase the chances of unwanted ribosome crushes. To further understand whether multidomain proteins might be prone to ribosome collisions, we used a well-established fluorescent reporter system. Initially, we replaced the poly-A stalling sequence with one, two and four Ig domains of the human Igλ light chain (IgL) (Figure 1a). Surprisingly, a single IgL domain interrupted translation to a similar extent to the poly-A cassette. On the other hand, two and four IgL domains did not affect the translation efficiency (Figure 1b). To reveal whether the observed translation inhibition is related to protein folding, we tested the effect of a structurally related Ig-like domain from Filamin A (FLNA) (Figure 1c). Due to their similar size and 3D arrangement, the presence of neither one nor multiple FLNA Ig-like repeats did not replicate the translation interruption caused by a single IgL domain (Figure 1d).

### 3.2. Translation of IgL Does Not Induce Ribosome Stalling

**Figure 2:**
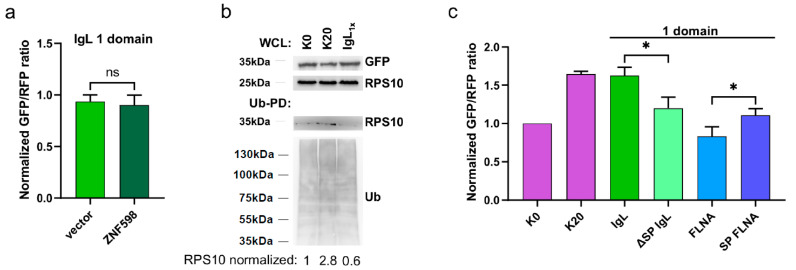
Evaluation of ribosome stalling and ER SP on translation efficiency of short mRNA. (**a**) Normalized GFP:RFP fluorescence ratio of the translation efficiency reporter containing a single repeat of the IgLλ domain (IgL) together with co-expression of empty vector (light green) or ZNF598 (dark green). Significance was compared using the two-tailed Student’s *t*-test, ns = non-significant. Error bars represent the mean ± SD. (**b**) Ubiquitin pull-down (Ub-PD) and whole cell lysate (WCL) from a cell expressing the translation efficiency reporter containing the control (K)0 and stalling (K)20 cassettes (reference) and a single repeat of the IgLλ domain (IgL_1x_). (**c**) Normalized GFP:RFP fluorescence ratio of the translation efficiency reporter containing the control (K)0 and stalling (K)20 cassettes (purple), a single repeat of IgL (green) and FLNA Ig-like domains with deletion (ΔSP IgL) and insertion of SP (SP FLNA). Significance was compared using the two-tailed Student’s *t*-test; * *p* = 0.05, ns = non-significant. Error bars represent the mean ± SD.

To examine whether the observed phenomenon is mechanistically related to the canonical ribosome stalling pathway, we overexpressed the E3-ligase ZNF598, which can inhibit translation through ubiquitination of the small ribosome subunit RPS10. Interestingly, ZNF598 failed to block translation in our single IgL domain reporter system (Figure 2a). Complementary to this, we analyzed the ubiquitination of endogenous RPS10, which is one of the initial events triggered by stalled ribosomes. As expected, RPS10 ubiquitination increased upon expression of the reporter with the poly-A sequence (K)20 but not a single IgL domain, even in the absence of proteasome inhibitors (Figure 2b). These results suggest that the inhibition of translation mediated by one IgL domain is likely not connected to ribosome stalling. Given that there was no effect of the sequentially and structurally highly related FLNA Ig-like repeat, we speculated that the subcellular localization of particular mRNAs might play a role in translation efficiency. Therefore, we deleted the ER-destined signal peptide from IgL (ΔSP IgL) and fused the same signal sequence to the Ig-like domain of FLNA (SP FLNA). In accordance with our hypothesis, swapping the ER-targeting SP rescued the translation block, mediated by a single IgL domain, while inducing partial translation inhibition in the presence of one Ig-like domain from FLNA (Figure 2c). Together, our data indicate that the translation efficiency of short mRNAs containing ER-targeting SPs might be significantly diminished.

### 3.3. The Number of Short ER-Destined Proteins Does Not Correlate with the Respective mRNAs

**Figure 3:**
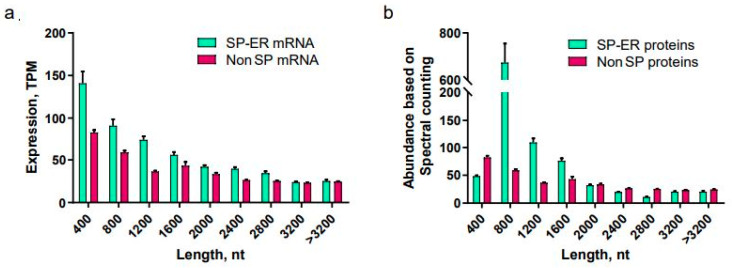
Analysis of transcript and protein abundance. (**a**) Average expression of human genes (transcripts per million reads) from 32 tissues in 122 individuals. Genes are divided into groups based on length of open reading frame. (**b**) Average abundance of human proteins (parts per million) and whole organism (integrated). Proteins are divided into groups based on length of relative coding sequence.

To determine whether the destination of translation to the ER might globally affect protein synthesis, we quantified the abundance of various mRNAs based on the presence of SPs. An analysis of the expression dataset [16,17] revealed a clear trend of enhanced transcription towards shorter mRNAs (Figure 3a). This tendency was particularly prominent in mRNAs encoding proteins with ER-targeting SPs. The absolute quantity of proteins mostly correlated with the relative amount of their respective mRNAs (Figure 3b) [18]. The exception was proteins whose corresponding mRNA open reading frame (ORF) was shorter than 400 nucleotides (nt). In this group, the number of synthesized proteins did not correlate with the relative number of matching mRNAs. This suggests that short mRNAs (ORF < 400 nt) containing ER-targeting SPs might indeed be translated less efficiently compared to longer mRNAs.

### 3.4. Length-Dependent Association of mRNA with Intracellular Membranes 

**Figure 4:**
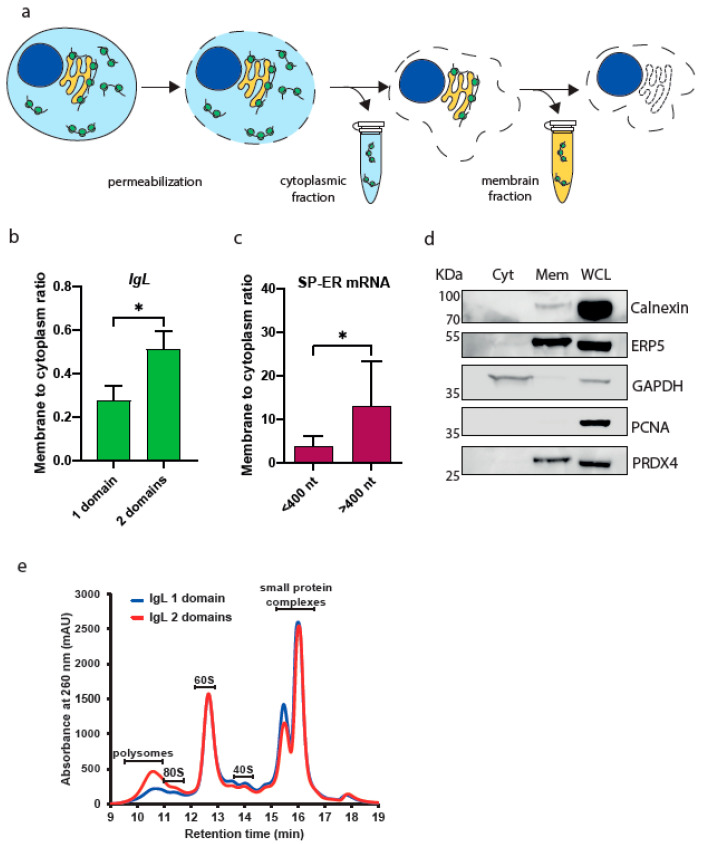
Membrane localization of short vs. long mRNAs. (**a**) Schematic representation of the fractionation assay. (**b**) The relative ratio of free (cytosolic) and membrane-bound (ER-associated) mRNAs encoding 1 and 2 IgLλ domains. Significance was compared using the two-tailed Student’s *t*-test; * = *p*, 0.05. Error bars represent the mean ± SD. (**c**) The relative ratio of free (cytosolic) and membrane-bound (ER-associated) endogenous mRNAs encoding short (<400 nt, *n* = 8 genes) and long (>400 nt, *n* = 9 genes) proteins containing ER-destined SPs. Significance was compared using the two-tailed Student’s *t*-test; * = *p*, 0.05. Error bars represent the mean ± SD. (**d**) WB analysis of fractionation samples. Cyt—cytosolic fraction, Mem—membrane fraction, WCL—whole cell lysate. GAPDH—cytosolic marker; calnexin, ERP5, PRDX4—ER markers; PCNA—nuclear marker. (**e**) Polysome analysis using Ribo Mega-SEC. Cell lysates from HEK293 cells transfected with plasmids encoding 1 and 2 IgLλ domains were separated using Agilent Bio SEC-5 2000 Å column. Retention time is indicated on the x-axis and UV absorbance at 260 nm is shown on the y-axis.

Given the apparent difference in the translation efficiency of short vs. long ER-destined proteins, we speculated that the length of an mRNA might affect the association of translation machinery with the ER membrane. To this end, we performed cell fractionation via sequential detergent extraction [15] to separate cytosolic and membrane-bound mRNAs (Figure 4a). The plasma membrane of the cells was permeabilized to allow for the collection of the cytosolic soluble fraction. After subsequent lysis, the soluble membrane fraction was collected. Initially, we used cells with the expression of single or tandem IgL domains. Upon quantification based on qPCR analysis of IgL mRNA, we found that the mRNAs encoding two IgL domains were more abundant in the membrane fraction compared to mRNAs with a single IgL domain ORF (Figure 4b). To validate this observation, we monitored the presence of endogenous mRNAs with ER-targeting SPs and monitored their presence in cytosolic and membrane pools based on the datasets analyzed in Figure 3, as well as mRNA expression normalized to transcripts per million (nTPM) values in the HEK293 cell line from the Human Protein Atlas [16]. The detected mRNAs were divided into two groups: short (>400 nt) and long (<400 nt) (see Supplementary Table S1). We found that the membrane-to-cytosol ratio was higher for longer mRNAs compared to short mRNAs (Figure 4c,d). These results indicate that the length of mRNAs with ER-destined SPs might define the interval of active translation on the ER membrane. 

The average distance between two elongating ribosomes on the eukaryotic mRNA is approximately 400 nt. Thus, we hypothesized that short mRNA (<400 nt) might be occupied with only one ribosome, while longer mRNA (>400 nt) contains multiple active translation complexes. To test this assumption, we analyzed polysome profiles from cells with overexpression of single and tandem IgL domains (Figure 4e). The cells with overexpression of mRNA encoding two IgL domains resembled enriched polysomal pools compared to cells expressing a single IgL ORF. This effect might at least partially be caused by highly abundant IgL mRNA transcribed from a strong promotor.

## 4. Discussion

The translation of the transmembrane, ER luminal, or secreted proteins starts in the cytosol, where the general translation factors and ribosomal subunits bind to mRNA. Once the SP is synthesized, the nascent polypeptide and ribosome–mRNA complex are recognized by SRP, which triggers a temporary pause in proteosynthesis. The SRP-dependent elongation arrest continues until proper localization of the translating ribosome on the ER membrane is ensured [19]. Moreover, translation arrest is crucial as SRP cannot target proteins when the nascent polypeptide exceeds a critical length [6]. The SRP-bound translation machinery engages with the SRP receptor on the ER membrane. The nascent polypeptide chain is positioned into a translocon, allowing for the active insertion of newly formed protein into the ER lumen. Meanwhile, SRP and SRP receptors are released from the translation complex [6].

The localization of the majority of mRNAs encoding SP-containing proteins targeted to the ER membrane is primarily mediated by the assembled translational complex that associates with SRP [19]. If the SP is present on the protein N-terminus, it is usually chopped off by ER-resident proteases soon after its insertion into the ER. Once the protein synthesis is complete, the ribosome dissociates from the mRNA thus allowing a new translational cycle to begin. Although the process is not fully understood yet, accumulating evidence also points to the existence of SP-independent targeting of the translation apparatus to the ER [20,21]. 

Translation efficiency can be regulated at various levels, including by the number of elongating ribosomes on a particular mRNA [22]. The minimal distance between actively translating ribosomes in eukaryotic cells is assumed to be around 400 nt [23]. Thus, the short mRNAs (ORF < 400 nt) encoding proteins directed to the ER can be occupied only by a single elongating ribosome at a time. Therefore, disassembly of the translation apparatus would likely lead to the dissociation of the short mRNA from the ER membrane to the cytosol. Re-initiation of the translation cycle will result in the synthesis of the SP sequence and the binding of SRP followed by a translation pause until the entire complex reaches the ER membrane. 

**Figure 5:**
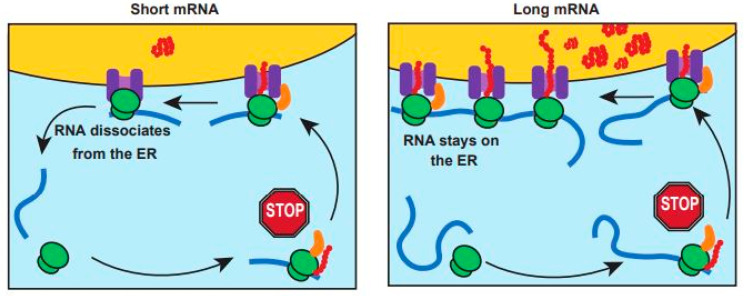
Schematic model of length-dependent translation efficiency of ER-destined proteins. The proposed model of SP-dependent localization of short vs. long ER-targeted mRNA (blue: mRNA, green: ribosome, orange: SRP, red: nascent polypeptide, purple: translocon, yellow: ER).

On the other hand, longer mRNAs coding for proteins with ER-targeting SPs (ORF > 400 nt) can be processed by multiple active ribosomes, forming a polysome assembly, that associates with the ER translocon at various elongation states. The continuous binding of translating ribosomes might allow for persisting localization of the long mRNAs on the ER membrane. Based on our data, we hypothesize that due to the proximity of the SRP-bound ribosomes with the respective translocons, the temporary translation pause caused by SRP binding is minimized. This ultimately results in increased efficiency of proteosynthesis from the longer mRNAs compared to the short mRNAs (Figure 5).

In accordance with our model, it was previously shown that transcripts with higher ribosome densities have a higher rate of translation. However, mRNAs with short ORFs are some of the most highly translated in the cell [24]. This apparent discrepancy is usually explained by the closed-loop model of the ribosome–mRNA translation complex. In this scenario, the eukaryotic mRNA is recruited to the ribosome via recognition of the 5′m7GpppN cap by translation initiation factor eIF4F, which can interact with the 3′poly(A)-binding proteins (PABP), bringing the two ends of the mRNA in close proximity [25]. 

Moreover, shorter mRNAs experience higher ribosome re-initiation rates due to higher enrichment with closed-loop factors, thus forming more closed-loop complexes and enabling the ribosomes to reinitiate translation on the same transcript multiple times [26]. It is therefore possible that short mRNAs contain a common attribute such as a *cis*-acting motif that might allow for the quick and preferential recruitment of closed-loop factors. Alternatively, a diffusion model was also suggested, as it would allow the end collision of shorter molecules, giving the closed loop more opportunities to form [27]. 

Another possibility is that ribosomes sense the length of ORFs, and thus, promote the interaction with closed-loop factors [2]. This length-dependent mechanism could have several advantages. Genes with the housekeeping role tend to be encoded in short ORFs [2,28] and require constant expression. Additionally, a closed loop would allow the cell to conserve resources in poor growth conditions [3]. In agreement with these studies, our work shows a higher abundance of shorter proteins.

These models, however, do not account for the specific subcellular localization of particular mRNAs, which could interfere with the interaction between translation initiation factors and PABP proteins. Specifically, the SRP-dependent delivery and subsequent association of the translation apparatus with proteins on the ER membrane might make it challenging to maintain the stability of the closed-loop structure. Indeed, we observed a drop in the abundance of a small pool of short SP-containing ER-targeted proteins (Figure 3). To fully delineate the mechanism behind the lower translation efficiency of short mRNAs that encode ER SP, additional studies are needed. Nevertheless, our findings provide potential new avenues to enhance the synthesis of small secreted proteins in eukaryotic expression systems.

## Data Availability

Not applicable.

## Supplementary Materials

The following supporting information can be downloaded at: https://www.mdpi.com/article/10.3390/cimb45080425/s1, Table S1: The list of mRNAs coding for the proteins with ER-targeting SP with short ORF (>400 nt) and long ORF (<400 nt) analyzed in cytosolic and membrane fractions in Figure 4c.
