# Supplementary material for: Length-Dependent Translation Efficiency of ER-Destined Proteins
Source: Curr Issues Mol Biol. 2023 Aug 14;45(8):6717–27. doi: 10.3390/cimb45080425 (PMC10453119; doi:10.3390/cimb45080425)
Supplement: Supplementary file 1 [file cimb-45-00425-s001.zip › cimb-2528252-supplementary.pdf]

Supplementary Table S1

mRNA <400 nt			
**name**	**code**	**length (bp)**	**nTPM**
Glucosidase II alpha subunit	GANAB	351	191.3
Glutamine amidotransferase like a class 1 domain containing 1	GATD1	306	120.5
Cellular repressor of E1A stimulated genes 1	CREG1	372	82.3
Glutaminyl-peptide cyclotransferase	QPCT	294	76
Haloacid dehalogenase-like hydrolase domain containing 5; Cat eye syndrome critical region protein 5	HDHD5	282	56.7
Store-operated calcium entry associated regulatory factor	SARAF	369	49.5
DiGeorge syndrome critical region gene 2; Integral membrane protein DGCR2/IDD	DGCR2	288	44.4
Small integral membrane protein 7 (isoform)	SMIM7	393	34.4
**mRNA >400 nt**			
**name**	**code**	**length (bp)**	**nTPM**
Peroxiredoxin 4	PRDX4	813	551.5
Selenoprotein F	SELENOF	1401	180.2
Galanin and GMAP prepropeptide	GAL	749	119.3
PTTG1 interacting protein	PTTG1IP	978	81
Microfibril-associated protein 2	MFAP2	897	78
Multiple coagulation factor deficiency 2, ER cargo receptor complex subunit	MCFD2	1143	67.2
Canopy FGF signaling regulator 2	CNPY2	1776	57.5
Serine peptidase inhibitor, Kunitz type 2	SPINT2	3378	51.7
Syndecan 2	SDC2	1167	44.4
